# Epidemiology and risk factors of needlestick injuries among healthcare workers in Iran: a systematic reviews and meta-analysis

**DOI:** 10.1186/s12199-021-00965-x

**Published:** 2021-04-01

**Authors:** Soheil Hassanipour, Mojtaba Sepandi, Reza Tavakkol, Mousa Jabbari, Hadiseh Rabiei, Mahdi Malakoutikhah, Mohammad Fathalipour, Gholamhossein Pourtaghi

**Affiliations:** 1 Faculty of Health, Baqiyatallah University of Medical Sciences, Tehran, Iran; 2 Health Research Center, Life Style Institute, Baqiyatallah University of Medical Sciences, Tehran, Iran; 3 Nursing and Midwifery Care Research Center, Mashhad University of Medical Sciences, Mashhad, Iran; 4 Department of Occupational Health and Safety, School of Public health and safety, Shahid Beheshti University of Medical Sciences, Tehran, Iran; 5 Department of Occupational Health Engineering, School of Public Health, Shahid Beheshti University of Medical Sciences, Tehran, Iran; 6 Occupational Health Engineering, Department of Occupational Health Engineering, School of Public Health, Kashan University of Medical Sciences, Kashan, Iran

**Keywords:** Needlestick injuries, Healthcare workers, Systematic review, Meta-analysis, Iran

## Abstract

**Background:**

Occupational contact with blood and body fluids poses a significant risk to healthcare workers. The aim of this systematic review is to investigate the epidemiology and risk factors affecting needlestick injuries (NSI) in healthcare personnel in Iran.

**Methods:**

In March 2020, researchers studied six international databases such as Medline/PubMed, ProQuest, ISI/WOS, Scopus, Embase, and Google Scholar for English papers and two Iranian databases (MagIran and SID) for Persian papers. Joanna Briggs Institute (JBI) Critical Appraisal Checklist was used to assess quality of studies. The method of reporting was based on the Preferred Reporting Items for Systematic Reviews and Meta-Analysis (PRISMA) statement.

**Results:**

A total of 43 articles were included in the analysis. Results showed that females (OR = 1.30, 95 % CI 1.06–1.58, *P* value = 0.009), younger age (OR = 2.75, 95 % CI 2.27–3.33, *P* value < 0.001, rotated shift workers (OR = 2.16, 95 % CI 1.47–3.15, *P* value < 0.001), not attending training courses (OR = 1.30, 95 % CI 1.07–1.56, *P* value = 0.006), working in the surgery ward (OR = 1.83, 95 % CI 1.33–2.50, *P* value < 0.001), less work experience (OR = 1.43, 95 % CI 1.04–1.95, *P* value = 0.025) apposed a greater risk factors for NSI among healthcare workers.

**Conclusion:**

Based on the results of this review, factors such as young age, less work experience, work shift, and female gender are considered as strong risk factors for NSI injury in Iran. Preventive measures including education programs can reduce the burden of NSI among healthcare personnel.

## Introduction

Needlestick injuries (NSI) are injuries caused by a needle head or a piece of broken ampule or other sharp object contaminated with blood or body secretions [1]. Occupational contact with blood and body fluids, followed by blood-borne infections, poses a significant risk to healthcare personnel [2]. At least 20 pathogenic pathogens can be transmitted following these injuries [3, 4]. Worldwide, about 25% of Hepatitis B virus (HBV) and Hepatitis C virus (HCV) infections and about 2.5% of HIV infections occur among healthcare workers due to NSI [5–7]. According to the World Health Organization, about 3 million out of the 35 million healthcare workers are exposed to NSI each year [8]. The annual economic burden of NSI was estimated to be $302 million in Japan [9]. The annual incidence of NSI was estimated at 20.5 per 1000 nurses and 16 per 1000 physicians in Poland. Overall, there were approximately 13,576 cases of NSI damage in Poland in 2014 [10].

Ghanei Gheshlagh et al.’s study showed the prevalence of needle head injury among healthcare personnel in Iran is 42.5%, and this rate is higher in females than males (47 vs. 42%) [11]. NSI-related risk factors have not yet been properly identified in Iran. Studies have identified factors such as excessive and unnecessary injections, poor personnel training, female gender, high workloads, and excessive fatigue especially at nighttime as the most important causes of NSI [7, 12–17]. Moreover, a systematic review found age, level of education, number of shifts per month, and history of training courses for individuals as factors influencing NSI. Several psychological problems in healthcare personnel are attributable to NSI that impose heavy costs on medical systems [18].

Many of NSIs are a source of infections are not reported due to fear of staff as well as lack of proper awareness. A few existing meta-analyses have only investigated the prevalence of NSI in medical personnel in Iran [19, 20]. Therefore, the aim of this systematic review is to investigate the epidemiology and risk factors affecting NSI in healthcare personnel in Iran.

## Methods

### Setting

The present study is a systematic review and meta-analysis of risk factors associated with NSI in medical personnel in Iran. The study was designed and conducted in 2020. The method of reporting the present study was based on the Preferred Reporting Items for Systematic Reviews and Meta-Analysis (PRISMA) statement.

### Search strategy

Six international databases including Medline/PubMed (http://www.ncbi.nlm.nih.gov), ProQuest (https://www.proquest.com/index), ISI/WOS (http://www.webofknowledge.com), Scopus (http://www.scopus.com), Embase (http://www.embase.com), and Google Scholar (https://scholar.google.com) were searched for English papers and two Iranian databases (MagIran [http://www.magiran.com] and SID [http://www.sid.ir]) for Persian papers from inception to March 2020. The selected keywords for databases included Needlestick OR Needle-stick OR Sharp Injury OR needle* stick injuries* OR injur* OR needlestick injur* OR sharp* OR injur* AND Iran". Two researchers reviewed reports independently.

### Study selection and data extraction

#### Inclusion and exclusion criteria

##### Inclusion criteria

The present study included only studies conducted in Iran and reported at least one factor affecting NSI.

##### Exclusion criteria

Studies without full text did not provide the information needed to enter the study and those that received a qualitative assessment score of less than 3.

### Definitions of some terms

*NSI:* Needlestick injury for at least 12 months

*Healthcare workers:* A healthcare worker is one who delivers care and services to the sick and ailing either directly. The majority of people in this group are nurses.

*Job stress:* Job stress is a type of stress caused by conditions in the workplace affecting a person’s performance. General Nordic questionnaire for psychological and social factors at work was used for assessing job stress.

### Quality assessment

The Joanna Briggs Institute (JBI) Critical Appraisal Checklist was used for quality assessment of included studies. This checklist examines the quality of cross-sectional studies. This checklist assesses 9 domains. The overall score above 7 indicates a high quality, between 4 and 6 shows medium quality, and below 3 shows poor quality.

### Screening of studies

The initial search was conducted by two researchers (X and Y). Study screening, extraction of results, and quality assessment were performed independently by two researchers (A and B). If there was no agreement between the two researchers, the team leader (C) would announce the final opinion on that article.

### Statistical analysis

The heterogeneity of the studies was investigated by Cochran’s test (with a significance level of less than 0.1) and its combination using *I*^2^ statistics (with a significance level greater than 50%). In case of model heterogeneity, random effects were used by variance image method, and in case of non-heterogeneity, the fixed effects model was used. The odds ratio (OR) index was used to combine results from different studies. This index provided the ability to combine studies that reported results in different ways. All analyses were performed by CMA statistical software version 2.

## Results

### Description of searched studies

**Fig. 1:**
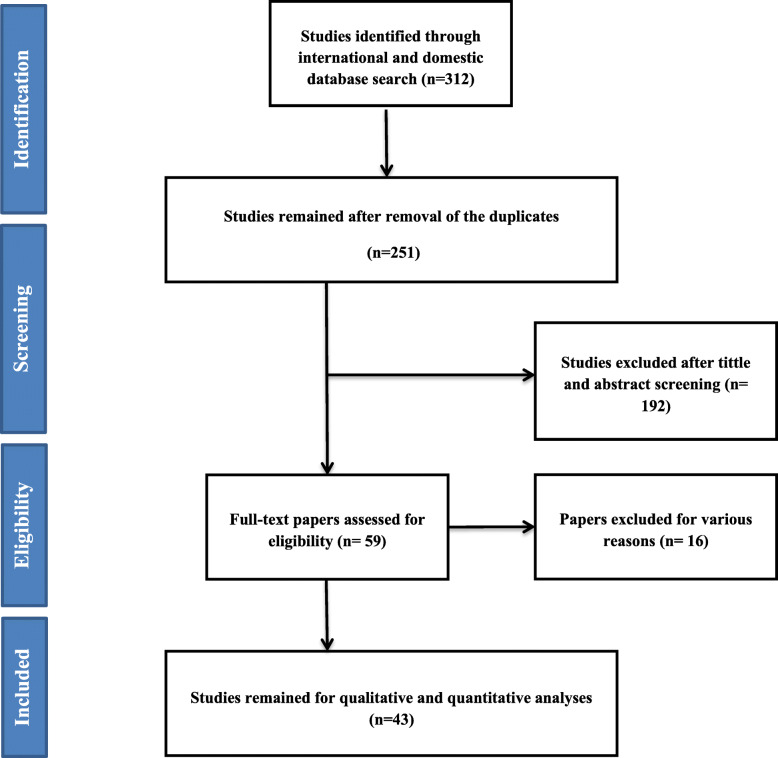
Flowchart of the included eligible studies in the systematic review

A total of 312 reports were found initially. After removing duplicates, 251 reports remained for title and abstract review. In total, 59 studies met the inclusion criteria and entered into the second stage of evaluation. Eventually, 43 studies were included in the final analysis. It should be noted that the references to the published articles were also reviewed to add relevant studies. Reasons for exclusion were unrelated topic (191), unrelated study population [13], and repetitive results [4]. The flowchart of the studies are presented in Fig. 1.

### Description of the included studies

Characteristics of the included studies [15, 21–64] are listed in Table 1.

**Table 1:** Descriptive data of included studies

Author, Year	Province	Design	Year of study	Population	Language	Sample size	Prevalence	Summary data for each study	Level of quality
Safaeian, 2019 [58]	Isfahan	CSS	2016	HCW	Persian	200	NR	Main risk factors related to NSI: Social support [odds ratio (OR) = 0.85, *P* < 0.001], psychosocial demand (OR = 1.07, *P* = 0.001), gender (OR = 0.44, *P* = 0.010), the dominant hand (OR = 0.52, *P* = 0.040), and participation in educational classes (OR = 0.24; *P* = 0.005)	High
Bagheri Hosseinabadi, 2019 [28]	Babol, Kerman, Mashhad, and Hamedan.	CSS	2015–2016	HCW	English	616	NR	Needlestick injuries showed a significant relation with age gender, work experience, and number of shifts in a month	High
Salmanzadeh, 2016 [59]	Dasht-e-Azadegan	CSS	2011	HCW	English	377	18.3	The highest frequency of needlestick injuries was observed at the night shifts (47.8%) and at the end of the shifts (64.70%).	Medium
Jahangiri, 2016 [15]	Shiraz	CSS	2014	HCW	English	168	76.0	A statistically significant relationship was found between the occurrence of NSIs and hours worked/week, sex, and frequency of shifts/month.	High
Mahmoudi, 2015 [48]	Tehran	CSS	2012	HCW	English	100	41.0	The relationship between occupational exposure to hospital sharp tools and age, experience, education, and place of work was significant with *P* = 0.006, 0.017, 0.027, and 0.008, respectively.	Medium
Izadi, 2015 [45]	Tehran	CSS	2011–2012	HCW	English	309	26.8	The incidence rate of NSIs in the HCWs on rotational shift work (90.4%) was higher than that among their counterparts on fixed shift work (9.6%).	Medium
Hajivandi, 2015 [40]	Bushehr	CSS	2013	HCW	Persian	68	58.8	Sharp injury events occurred more frequently at the hours from 11:00 to 14:00 and after 16:00.	Medium
Ghasemzadeh, 2015 [37]	Hormozgan	CSS	2012–2013	SN	English	377	39.3	A significant relationship was found between workplace and the NSI. Sixty-three (42.6%) of the injured students were interns in the emergency department (*P* value < 0.001).	Medium
Balouchi, 2015 [29]	Kerman	CSS	2014–2015	HCW	English	200	64.0	The results of Spearman’s correlation coefficient test showed no statistically significant relationships between the history of needlestick injuries and variables including work experience, age, and the number of shifts per month.	Medium
Mirzaei-Alavijeh, 2014 [50]	Kermanshah	CSS	2013	HCW	English	58	41.4	Logistic regression showed that sex (OR = 2.872) and job stress (OR = 1.503) could predict NSI.	Medium
Mehrdad, 2014 [49]	Tehran	CSS	2012	HCW	English	339	58.1	There is a significant association between increasing psychosocial factors at work and exposure to blood-borne pathogens.	High
Lakbala, 2014 [46]	Hormozgan	CSS	2013	HCW	English	215	89.3	The commonest reasons for non-compliance with NSI local protocols were not being sure of the local protocols (20.4%) and prolonged operation so unable to leave operation table (17.3%).	Medium
Ghanei Gheshlagh, 2014 [35]	Saqqez	CSS	2014	HCW	Persian	120	44.2	Comparing with other HCWs, those with needlestick injury were younger (*P* = 0.01) and had less work experience (*P* = 0.03).	Medium
Shoghli, 2013 [62]	Zanjan	CSS	2011	HCW	Persian	600	53.6	The frequency of NSI had a reverse relationship with age and work history, and it was significantly higher in male workers.	High
Rezaei, 2013 [57]	Tehran	CSS	2006–2009	HCW	English	514	26.0	There was no statistically significant difference in demographic variables except in work experience between two groups.	Medium
Gholami, 2013 [38]	Neyshabur	CSS	2011	HCW	English	384	32.0	Age (OR = 0.551, 95% CI 0.325–0.934) and number of shifts per month (OR=2.404, 95% CI 1.389–4.160) were found to be significantly associated with occurrence of needlestick and sharps injuries.	Medium
Adib-Hajbaghery, 2013 [22]	Kashan	CSS	2012	HCW	English	298	38.3	32.5% of injuries from sharp instruments occurred in the morning shift.	High
Ehsani, 2013 [33]	Tehran	CSS	2009	HCW	English	328	45.12	There were significant associations between the staff age as well as the ward with the extent of injuries.	Medium
Tirgar, 2012 [63]	Babol	CSS	2010	HCW	Persian	340	59.7	Statistical analysis showed that age, work experience, and received educational course could be associated with NSI.	Medium
Sharifian, 2012 [60]	Tehran	CSS	2008–2009	HCW	Persian	350	19.7	There was no statistically difference between job stress and NSI (*P* = 0.374).	Medium
Hashemi, 2012 [41]	Hamedan	CSS	2010	HCW	Persian	700	24.1	Statistical analysis showed that gender (female) and age group [30–34] could be associated with NSI.	Medium
Ghannad, 2012 [36]	Hamedan	CSS	2007–2008	HCW	English	89	NR	The most exposed age group was 25–34 years (51.6%).	Medium
Bijani, 2012 [30]	Qazvin	CSS	2009	HCW	Persian	246	31.3	Statistical analysis showed that work load could be associated with NSI.	Medium
Shiva, 2011 [61]	Tehran	CSS	2009	PHCP	English	355	49.3	Needlestick injuries are common among pediatric healthcare personnel, and their knowledge about prevention strategies is suboptimal.	Medium
Mohammadi, 2011 [51]	Qazvin	CSS	2008	HCW	English	138	52.9	The rate of NSI was significantly higher in the general surgery ward.	High
Bijani, 2011 [31]	Qazvin	CSS	2009	HCW	Persian	172	32.0	There was no statistically significant relationship between needlestick injuries and educational level, gender, and related training courses, but there was a statistically significant relationship between the injuries and the number of continuous shifts.	Medium
Azadi, 2011 [27]	Tehran	CSS	2009	HCW	English	111	45.9	Statistical analysis showed that gender (female), age group [26–30], and HBV vaccination could be associated with NSI.	Medium
Moradi, 2010 [52]	Bahar	CSS	2008	HCW	Persian	182	48.9	The risk of occupational injuries increased as the work experience increased (OR = 1.07, CI = 1.03–1.12). There was no significant relationship between employees' sex and age, and occupational injuries.	Medium
Heidari, 2010 [42]	Borujen and Lordegan	CSS	2007–2008	HCW	Persian	77	45.4	The needlestick exposure was not significantly different between males and females.	Medium
Gholami, 2010 [39]	Urmia	CSS	2008	HCW	Persian	400	26.7	Needlestick injuries in females and males were 28% and 24%, respectively. Most of the injuries were created by needle (47.3%) and anjiocat needle (19.9%).	Medium
Galougahi, 2010 [34]	Tehran	CSS	2008	HCW	English	158	56.9	There was no relationship between age, gender, years of professional life, and education level, and NSI.	Medium
Mohammadnejad, 2010 [43]	Tehran	CSS	2008	HCW	Persian	218	43.1	Statistical analysis showed that work experience could be associated with NSI.	Medium
Rakhshani, 2009 [56]	Zahedan	CSS	2007	HCW	Persian	231	64.9	Statistical analysis showed that education level and work experience could be associated with NSI.	High
Abdi, 2009 [21]	Jahrom	CSS	2006–2007	HCW	Persian	298	48.3	Most of the NSIs were related to HCWs with rotated working shifts..	Medium
Mohammadnejad, 2009 [44]	Tehran	CSS	2006	HCW	Persian	68	47.0	There were significant associations between the staff age as well as the work experience with the extent of injuries.	Medium
Jonaidi Jafari, 2008 [54]	Tehran	CSS	2007	HCW	Persian	613	32.7	There was significant associations between the workplace section and NSI.	Medium
Lotfi, 2008 [47]	Astara	CSS	2006	HCW	Persian	90	67.0	Multiple logistic regression analysis showed that the most important risk factor for needlestick injuries was working night shifts, (OR 2.5, 95% CI 1.5–4.8). Other important risk factor including lack of training on such injuries (OR 1.89, 95% CI 1.1–3.4), number of patients attended daily or nightly (OR 1.81, 95% CI 1.1–2.8), and recapping needles (OR 1.67, 95% CI 1.1–2.3).	Medium
Askarian, 2008 [24]	Fars	CSS	2006–2007	HCW	English	2118	35.2	NSIs were independently associated with gender, professional level, and hospital location.	High
Ebrahimi, 2007 [32]	Shahroud	CSS	2005	HCW	English	180	63.3	There were significant associations between the staff gender as well as the work experience and the extent of injuries.	Medium
Azadi, 2007 [26]	Tehran	CSS	2005	HCW	Persian	111	46.0	Statistical analysis showed that gender (female), age, and work experience was associated with NSIs.	Medium
Vahedi, 2006 [64]	Kurdistan	CSS	2004	HCW	Persian	847	43.5	There was significant associations between the work load and NSI.	Medium
Nejadrahim, 2005 [55]	Urmia	CSS	2004	HCW	Persian	434	52.5	57.3% of women and 45.3% of men had at least 1 event of NSI in the last year which showed a meaningful statistical difference (*P* value = 0.015).	Medium
Nazmieh, 2005 [54]	Yazd	CSS	2003–2004	HCW	Persian	1020	38.7	There were significant statistical correlations between the variables of the injuries and age, as well as the injuries with occupational groups and work settings (*P* = 0.04, 0.000, and 0.000), respectively.	Medium

*HCW* healthcare worker, *NSI* needlestick injury, *CSS* cross-sectional study, *SN* student nurse, *NR* not reported, *PHCP* Pediatric healthcare personnel

### Results of quality assessment

Eight studies were judged to have a high quality, and 35 had a medium quality.

### Results of heterogeneity

Results of the study heterogeneity for each of the risk factors are shown in Table 2.

**Table 2:** Results of heterogeneity among included studies

Variables	# of studies	***Q*** value	***I***^**2**^ (%)	***P*** value	Selected model
Hepatitis B vaccination status (Incomplete vs. complete)	4	9.9	69.9	0.019	Random
Employment status (Official vs. contract)	5	9.4	57.7	0.041	Random
Education level (< 12 years vs. > 12 years)	16	73.5	79.5	< 0.001	Random
Marital status (Single vs. married)	9	15.1	47.1	0.056	Fixed
Education level (< 16 years vs. > 16 years)	4	2.5	0.0	0.471	Random
Gender (females vs. males)	27	111.7	76.7	< 0.001	Random
Attending in training course (No vs. yes)	8	14.9	53.2	0.036	Random
Work experience (< 5 years vs. > 5 years)	5	10.8	63.2	0.028	Random
Job stress (severe vs. mild)	3	7.8	74.3	0.020	Random
Work experience (< 10 yeas vs. > 10 years)	10	37.6	76.0	< 0.001	Random
Age (< 30 vs. > 30)	13	69.6	82.7	< 0.001	Random
Shift working (night vs. day)	8	59.2	88.1	< 0.001	Random
Ward (surgical vs. medical)	11	68.8	85.4	< 0.001	Random
Shift working (rotate vs. fixed)	7	41.8	85.6	< 0.001	Random
Age (< 35 vs. > 35)	4	1.5	0.0	0.679	Fixed

### Results of meta-analysis

Results for NSI risk factors including gender, age, education level, employment status, job stress, and marital status are as follows:

#### Gender

**Fig. 2:**
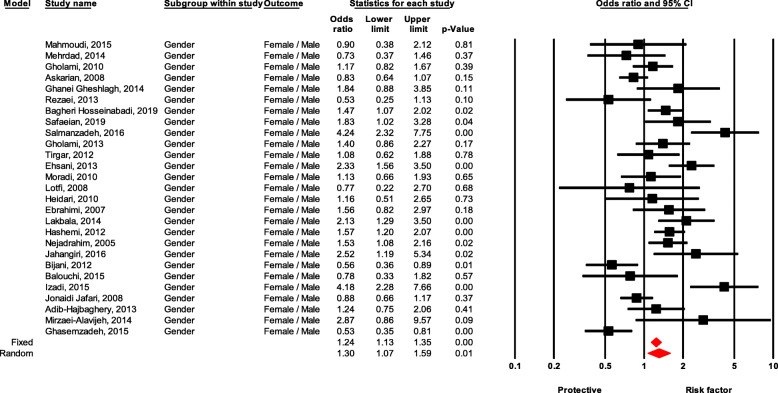
Forest plot for relationship between gender and risk of NSI in Iran

There was a significant difference between males and females experiencing NSI. Females had 30% more NSI experience than males (OR = 1.30, 95 % CI 1.06–1.58, *P* value = 0.009) (Fig. 2).

#### Age

**Fig. 3:**
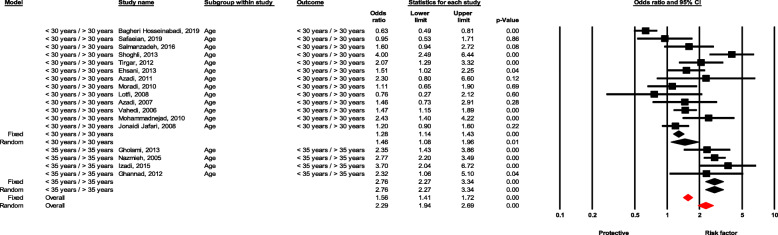
Forest plot for relationship between age and risk of NSI in Iran

Healthcare workers under the age of 30 had significantly higher likelihood of experiencing NSI than those over the age of 30 (OR = 1.45, 95 % CI 1.07–1.95, *P* value = 0.015), as well as healthcare workers under the age of 35 compared with those over the age of 35 (OR = 2.75, 95 % CI 2.27–3.33, *P* value < 0.001) (Fig. 3).

#### Education level

**Fig. 4:**
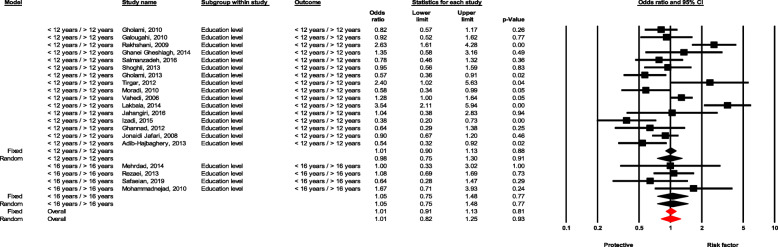
Forest plot for relationship between education level and risk of NSI in Iran

There was no significant difference between healthcare workers in the NSI event based on the two levels of education of 12 years (OR = 0.98, 95 % CI 0.74–1.29, *P* value = 0.887) and 16 years (OR = 1.05, 95 % CI 0.74–1.48, *P* value = 0.781) (Fig. 4).

There was no significant difference for NSI in terms of education level (OR = 0.98, 95 % CI 0.74–1.29, *P* value = 0.887; for 12 years education, and OR = 1.05, 95 % CI 0.74–1.48, *P* value = 0.781 for 18 years education) (Fig. 4).

### Employment status

**Fig. 5:**
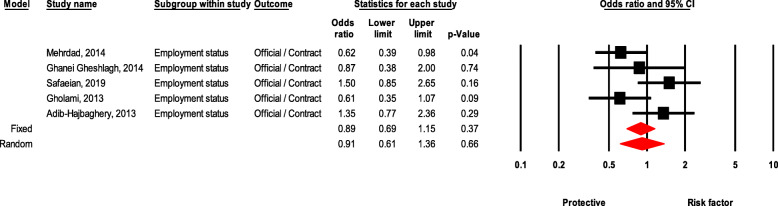
Forest plot for relationship between employment status and risk of NSI in Iran

There was no significant difference for experiencing NSI between permanent healthcare workers with contractual workers (OR = 0.91, 95 % CI 0.60–1.35, *P* value = 0.645) (Fig. 5).

### Job stress

**Fig. 6:**
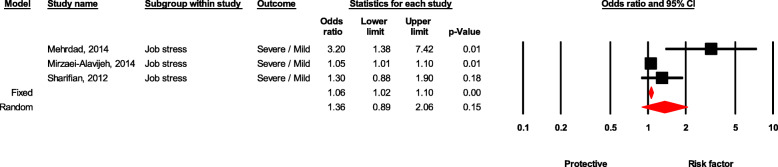
Forest plot for relationship between job stress and risk of NSI in Iran

The healthcare workers with severe job stress were 36% more likely to experience NSI than those with moderate stress, although it was not statistically significant (OR = 1.36, 95 % CI 0.89-2.08, *P* value = 0.151) (see Fig. 6).

### Marital status

**Fig. 7:**
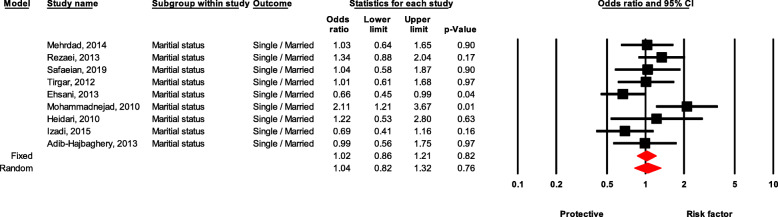
Forest plot for relationship between marital status and risk of NSI in Iran

There was no significant difference between singles and married healthcare workers in the NSI event (OR = 1.02, 95 % CI 0.86–1.21, *P* value = 0.820) (Fig. 7).

### Shift working

**Fig. 8:**
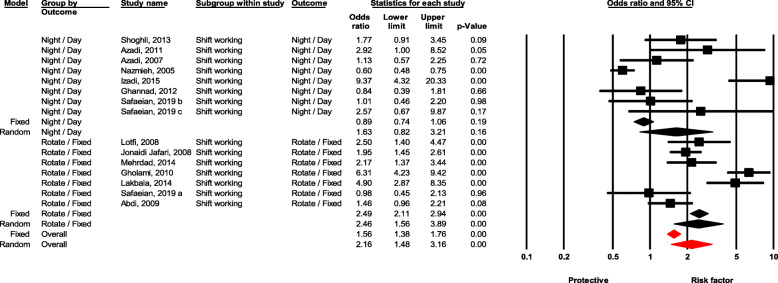
Forest plot for relationship between shift working and risk of NSI in Iran

Workers with rotating shifts were significantly more likely to experience NSI compared to fixed time workers (OR = 2.16, 95 % CI 1.47–3.15, *P* value < 0.001). Moreover, night-shift workers had higher likelihood of experiencing NSI compared with day-shift workers, but the difference was non-significant (OR = 1.63, 95 % CI 0.82–3.22, *P* value = 0.161) (Fig. 8).

### Attending in training course

**Fig. 9:**
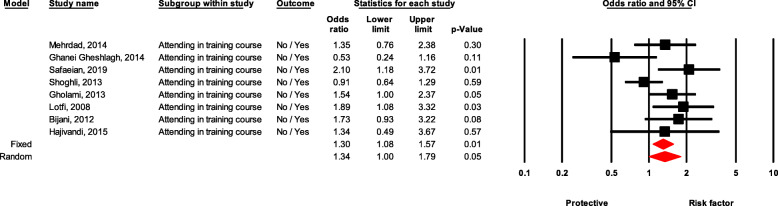
Forest plot for relationship between attending in training course and risk of NSI in Iran

Healthcare workers who did not attend the training courses were significantly 30% more likely to experience NSI than those who did the training (OR = 1.30, 95 % CI 1.07–1.56, *P* value = 0.006) (Fig. 9).

### Hepatitis B vaccination status

**Fig. 10:**
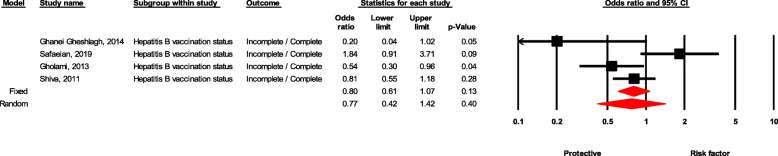
Forest plot for relationship between hepatitis B vaccination status and risk of NSI in Iran

Workers with incomplete vaccination against hepatitis B were 23% less likely to experience NSI than those who were fully vaccinated, although non-significantly (OR = 0.77, 95 % CI 0.41–1.41, *P* value = 0.400) (Fig. 10).

### Ward

**Fig. 11:**
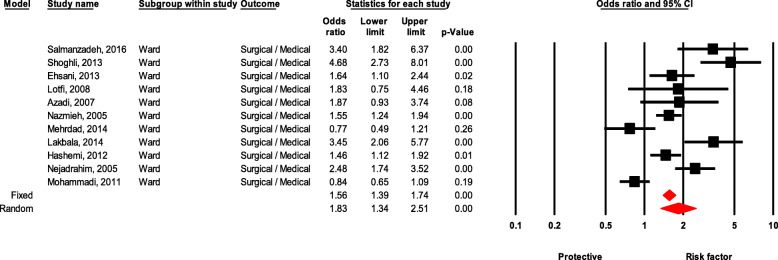
Forest plot for relationship between ward in hospital and risk of NSI in Iran

Healthcare workers in the surgery department were 83% more likely to have NSI than workers in the medical department, which was statistically significant (OR = 1.83, 95 % CI 1.33–2.50, *P* value < 0.001) (Fig. 11).

### Work experience

**Fig. 12:**
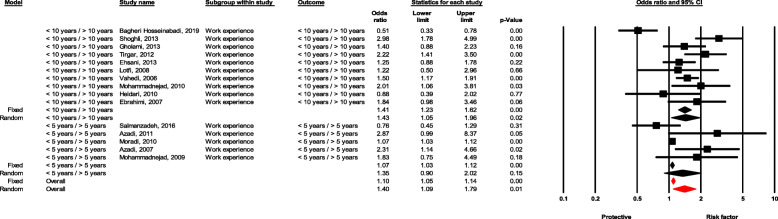
Forest plot for relationship between Work experience and risk of NSI in Iran

Healthcare workers with less than 10 years of experience were 43% more likely to have NSI than workers with more than 10 years of experience, which was statistically significant (OR = 1.43, 95 % CI 1.04–1.95, *P* value = 0.025). Moreover, healthcare workers with less than 5 years of experience had 35% higher chance of NSI than those with more than 5 years of experience, although the difference was not significant (OR = 1.35, 95 % CI 0.90–2.02, *P* value = 0.146) (Fig. 12).

**Fig. 13.**
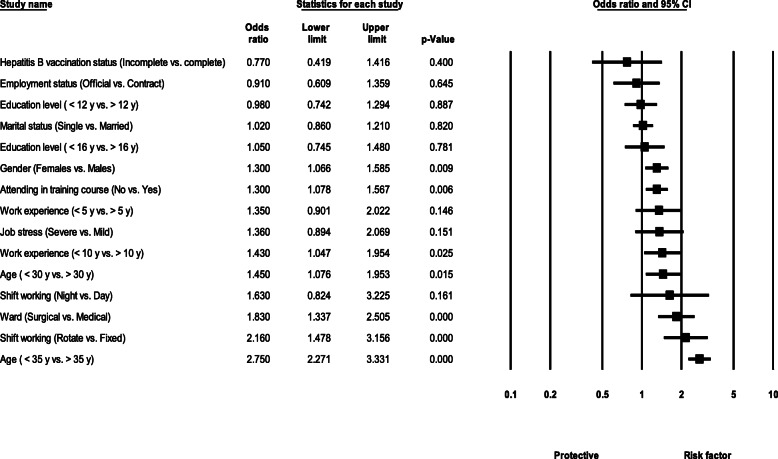
Summary of associated factors related to NSI in HCW in Iran

Summary of risk factors associated with NSI among HCW in Iran is presented in Fig. 13.

### Publication bias

**Fig. 14:**
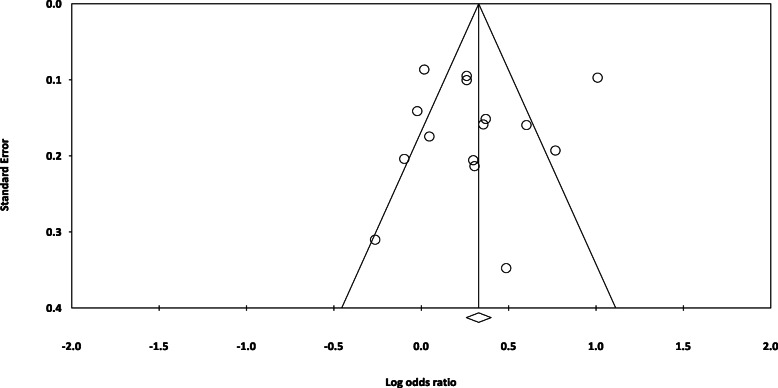
Funnel plot for assessing the risk of publication bias

The results of the Egger (*P* = 0.737) and Begg test (*P* = 0.552) revealed no evidence of publication bias. The funnel plot for assessing publication bias is shown in Fig. 14.

## Discussion

The aim of this systematic review and meta-analysis was to investigate the risk factors of NSI among healthcare workers in Iran. A number of risk factors associated with NSI have been identified. Factors such as female gender, younger age, work experience, job stress, work shift, education, and hospital ward were found to have a significant influence on the incidence of NSI.

Females are about 30% more likely to experience NSI injury than males. This might be due to various factors including stress and mental conflicts especially in the context of Iran as women have multiple roles in the home and workplace. Previous studies by Marawan Gabr et al., Teju Legesse et al., and Abimbola Oluwatosin et al. [65–67] are inconsistent since they identified males are more likely than females to experience NSI. It seems that the occurrence of NSI in terms of gender cannot be judged with confidence.

Furthermore, age and work experience of healthcare workers were identified as important factors related to NSI. Health workers under 30 years were more likely (about 50%) to have NSI than workers over 30 years. In addition, workers with a work experience of less than 10 years were nearly twice as likely to be affected by NSI. Occupational accidents occur more among inexperienced HCWs than experienced counterparts. Reasons might include unfamiliarity with the work environment and work process, less training, less risk awareness, and lack of experience with the same accident (for her/himself or others). Similar reasons can be found in medical staff. The results of the present study showed that education is an effective factor, inexperienced and young staff receive less training than experienced staff. Tolesa Bekele et al. [68] found that HCWs under 30 years of age suffered from NSI almost twice as much as staff aged over 30 years. In a study by Marawan Gabr et al. [65], staff with less than 15 years of work experience were more likely to have NSI compared with staff with more work experience. In another study, Abimbola Oluwatosin et al. [67] found a significant association between age of staff and the incidence of NSI, where staff in the age group of 25 years and younger were more likely to have NSI than the age group of 46 and older. Similar findings have been reported in Rajput et al.’s study among nurses. Such studies also mentioned insufficient training and other factors mentioned above as the reason for more NSI in younger and less experienced staff.

Type of hospital ward showed a significant effect on the incident of NSI. Surgical ward posed a higher risk on the incident of NSI incident compared with other departments. In a similar vein, Marawan Gabr et al. [65] found the incidence of NSI in the surgical ward was significantly higher than the medical ward. Moreover, the most common injuries related to NSI occurred in the emergency department in two studies by Varun Goel et al. [7] and Tolesa Bekele et al. [68]. The high incidence of NSIs in the surgery and emergency wards might be caused by high levels of stress resulting from exposure to high-risk patients, work sensitivity, and the need for extreme attention to patients which in turn reduces the nurses’ focus during work and increases NSI likelihood.

Information about the type of healthcare profession in our study was limited. In Gańczak et al.’s study, being a doctor was associated with greater odds (OR = 4.2) of suffering from injures in surgical wards versus nurses [69]. A systematic review and meta-analysis by Bouya et al. [70] about job category and NSIs show that prevalence of NSIs was highest among dentists (59.1%). The prevalence of NSIs in other occupational groups was 42.8% for nurses, 46.4% for physicians, and 45.3% for nursing students [70]. On the other hand, in some studies, nurses are considered to have high risk of NSIs compared with other groups [71, 72].

The present review found that Job stress is likely to increase NSI incident. Although no significant difference was found between severe and moderate job stress, staff with higher levels of stress had 36% more chance to experience NSI. In a study by Dilie et al., they showed that almost half of the staff with job stress experienced NSI [73]. Job stress can affect workers’ physical, physiological, and psychological responses and, in turn, their mental, physical, or emotional activities leading to more mistakes during work and reduced work performance [74]. Consequently, NSIs are quite likely among staff with higher job stress.

The present review showed that staff with rotational and night shifts are more likely to have NSI than others. Similar to the present findings, Marawan Gabr et al. showed that night shifts increase NSI likelihood. They found that staff with more than 2 night shifts per month were more likely to experience NSI [65]. One possible explanation could be the changes in body's natural physiological cycle as it is related to stress and NSIs. However, in a non-aligned study, Kasatpibal et al. found that most NSIs for nurses in the surgery rooms occurred during morning shifts. This is also justified by the high workload of nurses (e.g., surgeries) in the morning shift [75]. Therefore, it seems that the incident of NSIs might depend on the workplace situation and job type.

Training courses were also identified as one of the key factors affecting the occurrence of NSIs. The incidence of NSIs was shown to be approximately one third (30%) in those who took training courses. In a study by Kasatpibal et al. [75], the effect of training with and without practicing on the incidence of NSIs was assessed. Staff who had training without practicing were significantly (about 53%) more likely to experience NSI. In another study evaluating the effect of attendance in training sessions, staff who did not attend the training sessions suffered more NSIs than staff who attended training [65]. On-job training can play a crucial role in increasing their performance and reducing job risks due to being in a clinical environment and direct exposure to risk factors. For this reason, in various studies, trained staff were less likely to face occupational hazards and injuries. These cases show a direct impact of clinical education on staff’s performance and NSI incident.

### Strengths and limitations

Previous meta-analyses conducted in Iran have estimated the prevalence of NSIs. However, the present study aimed to estimate the risk factors of NSI. Two limitations of this review were the lack of detailed information about the type of healthcare professions and the content of training packages.

### Recommendations

It is recommended that healthcare authorities plan regular training programs for the prevention of NSIs in healthcare workers. In order to improve these training methods, staff evaluation should be done in different time periods, and possible mistakes should be corrected. Other recommendations are to establish uniform policies across all hospitals about the management of NSIs and performing periodic practical and verbal exams on personnel knowledge, attitude, and performance regarding prevention of NSIs.

## Conclusions

In conclusion, this review identified key risk factors including young age, less work experience, work shift, and female gender for NSIs in Iran. Preventive activities based on known risk factors can reduce the burden of NSI on healthcare personnel.

## Abbreviations

HCW: Health-care workers
NSI: Needlestick injuries
